# Resurgence of respiratory syncytial virus infection during COVID-19 pandemic in Pune, India

**DOI:** 10.1186/s12879-024-09426-6

**Published:** 2024-06-14

**Authors:** Sumit Bhardwaj, Manohar Lal Choudhary, Mandeep S Chadha, Aarti Kinikar, Ashish Bavdekar, Nilesh Gujar, Pradeep dcosta, Rajesh Kulkarni, Sanjay Bafna, Sonali Salvi, Vikram Padbidri, Varsha Potdar

**Affiliations:** 1 Influenza, National Institute of Virology (ICMR), Pune, India; 2 B.J.Government Medical College and Sassoon General Hospital, Pune, India; 3 KEM Hospital Research Centre, Pune, India; 4 Gujar Children Hospital, Pune, India; 5 Microbiology, Jehangir Hospital, Pune, India

**Keywords:** Respiratory syncytial virus, RSV subtypes, COVID-19 pandemic, Prevalence, Co-infections, Symptomatology, Therapeutic interventions

## Abstract

**Introduction:**

Respiratory Syncytial Virus (RSV) is a leading cause of acute lower respiratory infection in children worldwide. Understanding its prevalence, variations, and characteristics is vital, particularly in the context of the COVID-19 pandemic.

**Objective:**

The study aimed to investigate the RSV positivity rate, subtype prevalence, age and gender distribution, symptomatology, and co-infection rates during pre-pandemic and pandemic periods.

**Methods:**

We analyzed data from 15,381 patients tested for RSV between 2017 and 2023.

**Results:**

Our analysis revealed a 7.2% average RSV positivity rate in the pre-pandemic period, with significant fluctuations during the pandemic (1.5% in 2020 to 32.0% in 2021). We observed variations in RSVA and RSVB detection rates. The 0–4 years’ age group was consistently the most affected, with a slight male predominance. Fever and cough were common symptoms. Therapeutic interventions, particularly antiviral usage and ventilation requirements, decreased during the pandemic. We also identified variations in co-infection rates with other respiratory viruses.

**Conclusion:**

Our study offers critical insights into the impact of the COVID-19 pandemic on RSV prevalence, subtype distribution, patient characteristics, and clinical management. These findings underscore the need for ongoing surveillance and adaptive public health responses.

## Introduction

The COVID-19 pandemic has profoundly influenced public health measures globally, resulting in substantial alterations in the epidemiology of various infectious diseases [1]. Implementation of nonpharmaceutical interventions (NPIs), such as lockdowns, social distancing, and masking, has inadvertently impacted the transmission of other respiratory pathogens, including respiratory syncytial virus (RSV) [2]. RSV is a primary cause of severe respiratory illness in young children and the elderly, contributing to significant morbidity and mortality [3]. Following the relaxation of COVID-19-related public health measures, several countries reported an intersessional resurgence of RSV [4, 5]. The altered transmission dynamics of RSV during the pandemic have raised concerns about the potential for a more significant resurgence post-pandemic [6, 7]. Recent studies have reported RSV co-infections with SARS-CoV-2 in hospitalized children, adding complexity to the management of these respiratory infections [8]. The resurgence of RSV has been reported in various countries, including Australia [4], the United States [9], and several Southeast Asian nations [10–12]. However, there is limited data on the post-pandemic RSV resurgence in India, a country with a significant burden of respiratory infections in children. This research article aims to explore the resurgence of RSV in Pune, India following the relaxation of COVID-19-related public health measures.

## Methods

The National Influenza Center at the Indian Council of Medical Research-National Institute of Virology conducted an investigation into the epidemiology and laboratory diagnosis of respiratory syncytial virus (RSV) in Pune, Western India, from January 2017 to February 2023. The study aimed to analyze the variations in RSV infection patterns during and prior to the COVID-19 pandemic. The designated ICMR-NIV institutional ethics committee approved all methods in this study and protocols, and informed consent was obtained from all subjects or their legal guardians, as applicable. With the approval of the institutional human ethics committee, participants were recruited from sentinel hospitals and clinics in Pune, India. In selecting the sentinel hospitals for this study, we focused on ensuring a comprehensive and representative sample of the population. To this end, both tertiary and secondary care hospitals were chosen, representing both the public and private sectors. This approach allowed us to capture a wide range of demographic and clinical data, essential for a thorough analysis of RSV patterns across different healthcare settings and patient populations. A total of six sentinel sites were included in this network. The selection of these sites was based on several factors, including their geographical location, patient volume, and the ability to provide high-quality clinical and laboratory data. This network of sentinel hospitals is crucial for our ongoing surveillance efforts and provides valuable insights into the epidemiology of respiratory viruses, including RSV. Acute respiratory infection (ARI) was characterized as cases in the outpatient department (OPD) exhibiting an acute onset (within 7 days) of at least two symptoms: fever/feverishness, chills, cough, nasal congestion, shortness of breath, or sore throat. Modified severe acute respiratory infection (SARI) was identified in patients with a cough beginning within the past seven days and requiring overnight hospitalization. In infants younger than two months, SARI was diagnosed as an acute lower respiratory infection necessitating hospitalization. Each week, 10–20 ARI and SARI patients across all age groups were recruited from sentinel hospitals. During periods of heightened respiratory illness in the community or hospital, additional individuals were included using convenient sampling. At sentinel locations, physicians and nurses were trained to screen patients based on ARI and SARI case criteria. Trained personnel collected respiratory specimens [Throat and nasal swab] (depending on the patient’s age i.e. younger than 5 years nasal swab taken while for older than 5 years both nasal and throat swab was taken) and transported them to the laboratory within 24 h while maintaining a cold chain. RNA extraction was performed using a MagMax-96 kit, and specimens were tested for RSV and other viruses using CDC real-time reverse transcription polymerase chain reaction (rRT-PCR) [13]. The study period was categorized into pre-pandemic (January 2017 to January 2020) and pandemic (February 2020 to February 2023) phases. Epidemiological weeks and seasonality were calculated based on the day of illness onset. Weekly data were compiled and presented as the percentage positive for each virus throughout an epidemiological year.

## Results

Of the 15,248 patients included in the study, 14,327 (93.2%) 1,054 (6.8%) tested positive. Table 1 Our sample included 9,812 patients during the pandemic and 5436 in the pre-pandemic period. During the pre-pandemic years, we observed an average RSV positivity rate of 7.3%, with 398 individuals testing positive for RSV out of 5,436 who were tested. This scenario underwent considerable fluctuations during the course of the COVID-19 pandemic. In 2020, the RSV positivity rate drastically dropped to 0.5% with only 16 individuals tested positive out of 3,169 tests conducted. This trend saw a remarkable rebound in 2021, with the positivity rate soaring to 23.2% (341 out of 1,471). This was followed by a 5.5% (212 out of 3,803) in 2022 and further to 6.6% (85 out of 1,369) positivity in 2023. The data shows significant variations in the detection rates of Respiratory Syncytial Virus A (RSVA) and B (RSVB) during both pre-pandemic and pandemic years. In the pre-pandemic period, both Respiratory Syncytial Virus A (RSVA) and B (RSVB) showed variation in subtype circulation and trends. RSVA accounted for a modest proportion of the total cases each year, ranging from 0.3% in 2016 to a peak of 23% in 2019. Interestingly, the annual RSVB detection rate was mostly higher than that of RSVA, except in 2019. The highest positivity rate for RSVB was observed in 2018 at 34%. In the year 2020, coinciding with the start of the COVID-19 pandemic, both RSVA and RSVB prevalence decreased dramatically to 2.7% and 0.2% respectively. The year 2021 saw a significant surge in RSVA cases, reaching a high of 58%. Remarkably, RSVB cases were not detected at all during this year. In 2022, the RSVA prevalence declined to 14% while RSVB re-emerged with a prevalence of 28%.

**Table 1:** Clinical characteristics and outcome of sample tested for RSV

	Pre-pandemic (2016-Jan 2020)		Pandemic 2020		Pandemic 2021		Pandemic 2022		Pandemic 2023	
	RSV Positive	Tested	RSV Positive	Tested	RSV Positive	Tested	RSV Positive	Tested	RSV Positive	Tested
	*N* = 398	*N* = 5,436	*N* = 16	*N* = 3,169	*N* = 341	*N* = 1,471	*N* = 212	*N* = 3,803	*N* = 85	*N* = 1,369
RSV Positivity	7.3%		0.5%		23.2%		5.5%		6.2%	
Female	169 (42%)	2,360 (43%)	7 (44%)	1,211 (38%)	140 (41%)	659 (45%)	97 (46%)	1,628 (43%)	33 (39%)	617 (45%)
Male	229 (58%)	3,076 (57%)	9 (56%)	1,958 (62%)	201 (59%)	812 (55%)	115 (54%)	2,175(57%)	52 (61%)	752 (55%)
Age cat(yrs)										
0 to 4	322 (81%)	2,065 (38%)	14 (88%)	312 (9.8%)	297 (87%)	672 (46%)	182 (86%)	1,732 (46%)	63 (74%)	577 (42%)
5 to 14	35 (8.8%)	1,106 (20%)	0 (0%)	154 (4.9%)	29 (8.5%)	219 (15%)	17 (8.0%)	756 (20%)	11 (13%)	319 (23%)
15 to 29	15 (3.8%)	708 (13%)	0 (0%)	520 (16%)	8 (2.3%)	156 (11%)	2 (0.9%)	213 (5.6%)	2 (2.4%)	92 (6.7%)
30 to 59	15 (3.8%)	1,035 (19%)	1 (6.2%)	1,355 (43%)	4 (1.2%)	241 (16%)	7 (3.3%)	603 (16%)	6 (7.1%)	176 (13%)
60+	11 (2.8%)	522 (9.6%)	1 (6.2%)	828 (26%)	3 (0.9%)	183 (12%)	4 (1.9%)	499 (13%)	3 (3.5%)	205 (15%)
Pregnant	7 (1.8%)	105 (1.9%)	0 (0%)	5 (0.2%)	0 (0%)	3 (0.2%)	0 (0%)	1 (< 0.1%)	0 (0%)	1 (< 0.1%)
Fever_H7days	363 (91%)	4,693 (86%)	16 (100%)	2,711 (86%)	284 (83%)	924 (63%)	197 (93%)	3,174 (83%)	76 (89%)	1,126 (82%)
Cough	364 (91%)	4,816 (89%)	16 (100%)	2,387 (75%)	298 (87%)	842 (57%)	190 (90%)	2,760 (73%)	72 (85%)	1,029 (75%)
Rigors	26 (6.5%)	1,047 (19%)	2 (12%)	32 (1.0%)	7 (2.1%)	71 (4.8%)	6 (2.8%)	302 (7.9%)	6 (7.1%)	139 (10%)
Sore throat	107 (27%)	2,693 (50%)	2 (12%)	567 (18%)	125 (37%)	402 (27%)	22 (10%)	754 (20%)	10 (12%)	244 (18%)
Breathlessness	244 (61%)	2,906 (53%)	15 (94%)	1,310 (41%)	129 (38%)	430 (29%)	114 (54%)	1,663 (44%)	48 (56%)	559 (41%)
Vomiting	91 (23%)	1,420 (26%)	7 (44%)	260 (8.2%)	94 (28%)	271 (18%)	19 (9.0%)	688 (18%)	9 (11%)	245 (18%)
Chest Pain	15 (3.8%)	615 (11%)	0 (0%)	94 (3.0%)	2 (0.6%)	27 (1.8%)	3 (1.4%)	222 (5.8%)	6 (7.1%)	73 (5.3%)
Body ache	53 (13%)	1,731 (32%)	2 (12%)	678 (21%)	16 (4.7%)	191 (13%)	10 (4.7%)	743 (20%)	6 (7.1%)	223 (16%)
Headache	31 (7.8%)	1,476 (27%)	2 (12%)	42 (1.3%)	5 (1.5%)	120 (8.2%)	4 (1.9%)	474 (12%)	6 (7.1%)	171 (12%)
Seizures	18 (4.5%)	205 (3.8%)	0 (0%)	3 (< 0.1%)	12 (3.5%)	66 (4.5%)	9 (4.2%)	281 (7.4%)	2 (2.4%)	103 (7.5%)
Abdominal pain	16 (4.0%)	498 (9.2%)	1 (6.2%)	87 (2.7%)	9 (2.6%)	53 (3.6%)	3 (1.4%)	250 (6.6%)	4 (4.7%)	104 (7.6%)
Diarrhea	29 (7.3%)	398 (7.3%)	0 (0%)	74 (2.3%)	25 (7.3%)	68 (4.6%)	6 (2.8%)	229 (6.0%)	8 (9.4%)	105 (7.7%)
Wheeze	195 (56%)	1,672 (47%)	12 (75%)	130 (5.5%)	70 (33%)	174 (15%)	89 (42%)	969 (25%)	35 (41%)	321 (23%)
Crepitation	216 (62%)	1,982 (56%)	13 (81%)	240 (10%)	83 (39%)	224 (19%)	65 (31%)	1,026 (27%)	24 (28%)	294 (21%)
Asthma	10 (2.5%)	117 (2.2%)	0 (0%)	28 (0.9%)	1 (0.3%)	12 (0.8%)	0 (0%)	84 (2.2%)	1 (1.2%)	27 (2.0%)
co-morbidities	0 (0%)	0 (0%)	0 (0%)	0 (0%)	0 (0%)	59 (4.0%)	13 (6.1%)	918 (24%)	9 (11%)	292 (21%)
antivirals	172 (43%)	1,972 (36%)	8 (50%)	168 (5.3%)	62 (18%)	183 (12%)	0 (0%)	143 (3.8%)	1 (1.2%)	2 (0.1%)
ventilation	40 (10%)	499 (9.2%)	0 (0%)	38 (1.2%)	16 (4.7%)	70 (4.8%)	0 (0%)	54 (1.4%)	0 (0%)	0 (0%)
steroid	75 (19%)	969 (18%)	0 (0%)	44 (1.4%)	20 (5.9%)	104 (7.1%)	1 (0.5%)	120 (3.2%)	0 (0%)	0 (0%)
ICU	178 (45%)	1,828 (34%)	6 (38%)	78 (2.5%)	27 (7.9%)	178 (12%)	12 (5.7%)	472 (12%)	10 (12%)	132 (9.6%)
Death	4 (1.1%)	175 (4.9%)	0 (0%)	1 (< 0.1%)	0 (0%)	2 (0.2%)	0 (0%)	0 (0%)	0 (0%)	0 (0%)

In the pre-pandemic timeframe, RSV showed a significant predilection for children aged 0 to 4 years, accounting for 322 out of 398 (81%) cases. Throughout the pandemic years, the pattern of RSV infection consistently remained skewed towards the 0–4 years age group, albeit the proportion fluctuated from a low of 74% (63 out of 85) in 2023 to a high of 87% (297 out of 341) in 2021. This reaffirms the known susceptibility of younger children to RSV. Pre-pandemic data shows a slightly higher prevalence of RSV among males (229 out of 398, 57.5%) than females (169 out of 398, 42.5%). During the pandemic years, the gender distribution among RSV positive cases showed little variation, with the proportion of males varying from 52 to 61% and females from 39 to 46%. No substantial gender disparity in RSV positivity was observed, implying a gender-independent transmission and susceptibility pattern to RSV.

In pre-pandemic period, acute respiratory infection (ARI) cases accounted for 35% (1,891 cases), while severe acute respiratory infection (SARI) cases represented 65% (3,545 cases) of the total respiratory infection cases. However, in the pandemic period, there was a slight shift in the distribution, with ARI cases comprising 27% (2,673 cases) and SARI cases making up 73% (7,139 cases) of the total respiratory infection cases. In terms of RSV positivity, during the pre-pandemic period, RSV was detected in 13% (50 cases) of ARI cases and in a higher proportion of 87% (348 cases) of SARI cases. This indicates a higher prevalence of RSV among severe respiratory infection cases in the pre-pandemic period. Conversely, during the pandemic, RSV positivity decreased to 33% (217 cases) among ARI cases, while it increased to 67% (437 cases) among SARI cases.

Fever and cough were ubiquitously reported among RSV positive cases across all years. Prior to the pandemic, 363 out of 398 (91%) cases reported a fever within the past 7 days, and a similar proportion, 364 (91%), reported a cough. These two symptoms remained the most common presentations during the pandemic years, although the prevalence varied slightly from 76 out of 85 (89%) to 284 out of 341 (83%) for fever and 72 out of 85 (85%) to 298 out of 341 (87%) for cough.

In terms of therapeutic interventions, the pre-pandemic era saw antiviral usage in 43% of cases (172 out of 398), and 10% required ventilation support (40 out of 398). However, the pandemic years witnessed a stark reduction in both parameters. Antiviral usage fell precipitously, with no usage reported in 2022 and minimal usage of 1.2% (1 out of 85) in 2023. Similarly, ventilation requirements were nonexistent in 2022 and 2023.

**Fig. 1:**
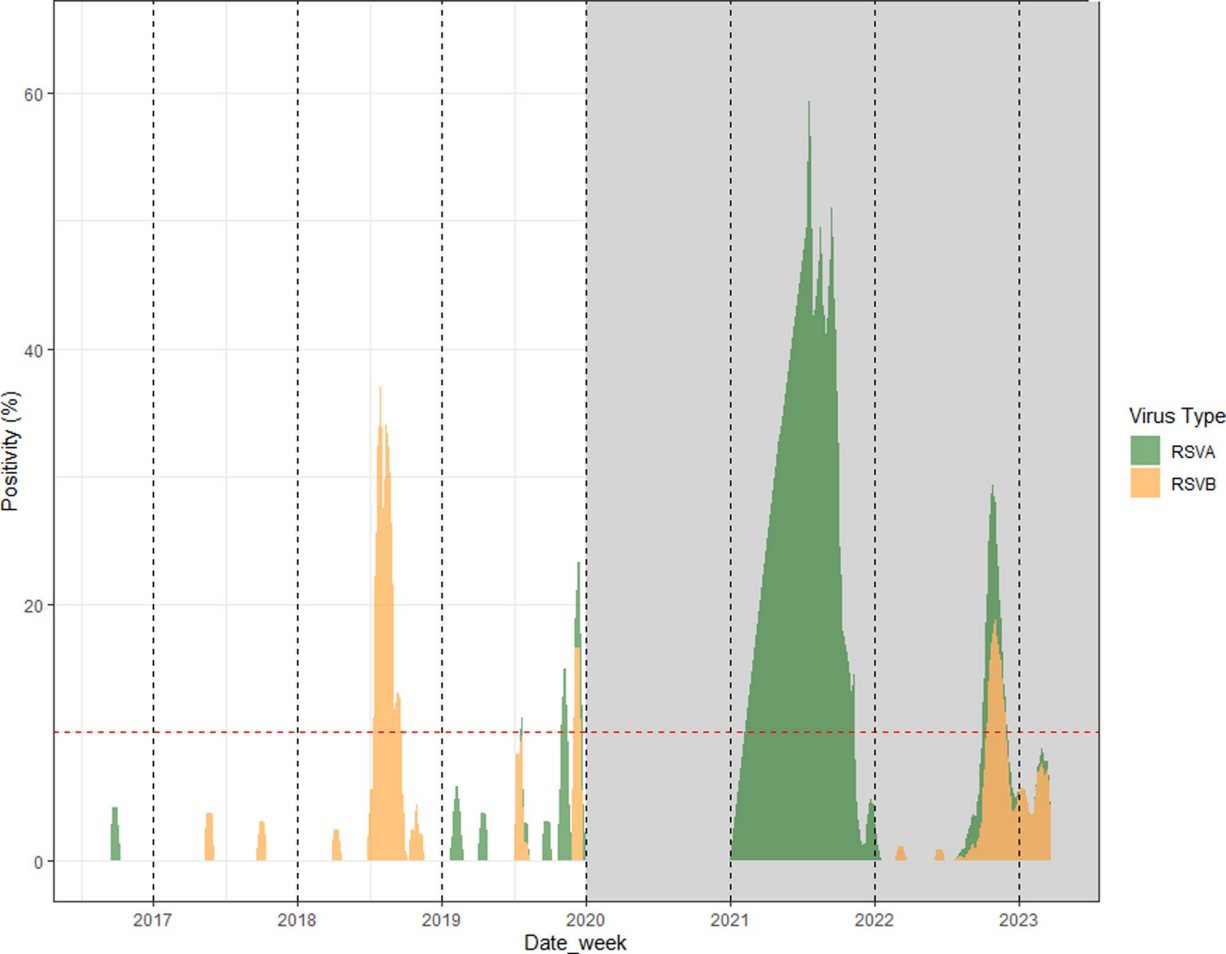
Three-week moving average of the RSV A and B positivity (2017–2023)

Figure 1 The pre-pandemic era witnessed several notable periods of peak activity of RSV -. For RSVA, there was one significant event. Between the weeks of 4th November 2019 and 18th November 2019, the RSVA positivity rate surged, signifying during a peak activity period. This incident is consistent with the increase in RSVA cases in 2019. During the same pre-pandemic era, RSVB also had two peak activity periods. The first surge of RSVB activity occurred between the weeks of 16th July 2018 and 24th September 2018. Subsequently, there was another notable period of increased RSVB activity from the weeks of 2nd December 2019 to 23rd December 2019. The timing of these RSVB peaks aligns with the substantial rise in RSVB cases in 2018 and the decrease towards the end of 2019. The years of the pandemic also saw several peak RSV activity periods. RSVA positivity surged twice during this time. The first notable increase was between the weeks of 3rd May 2021 and 15th November 2021. A second period of heightened RSVA activity took place from the weeks of 10th October 2022 to 31st October 2022For RSVB, there was single peak activity observed during the pandemic years, between the weeks of 10th October 2022 and 5th December 2022. This corresponds to the resurgence of RSVB cases during 2022. In summary, the pre-pandemic years saw one peak in RSVA activity and two in RSVB activity. In contrast, the pandemic years experienced two peaks in RSVA activity and one for RSVB. This data further corroborates the shifts in RSV subtype prevalence during the pandemic years as compared to the pre-pandemic era.

**Fig. 2:**
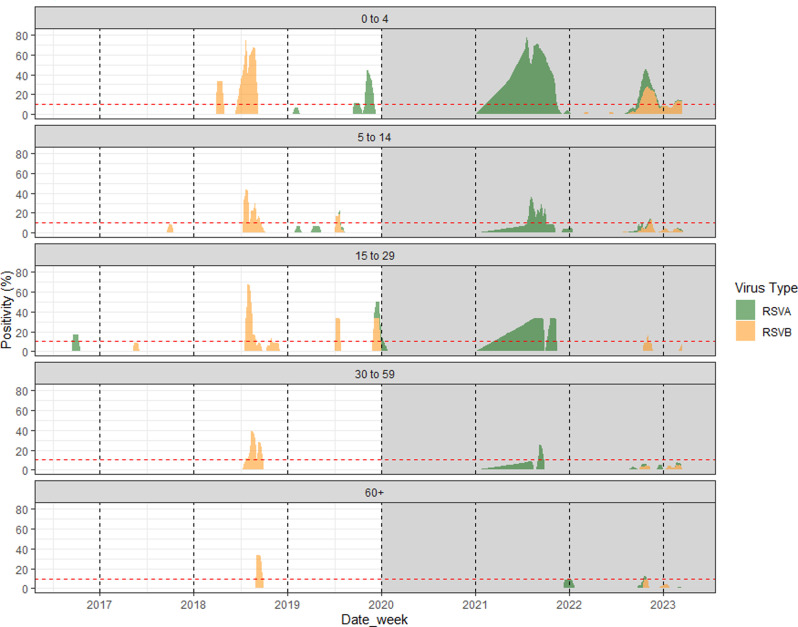
Three-week moving average of the RSV A and B positivity stratified by age groups (2017–2023)

**Fig. 3:**
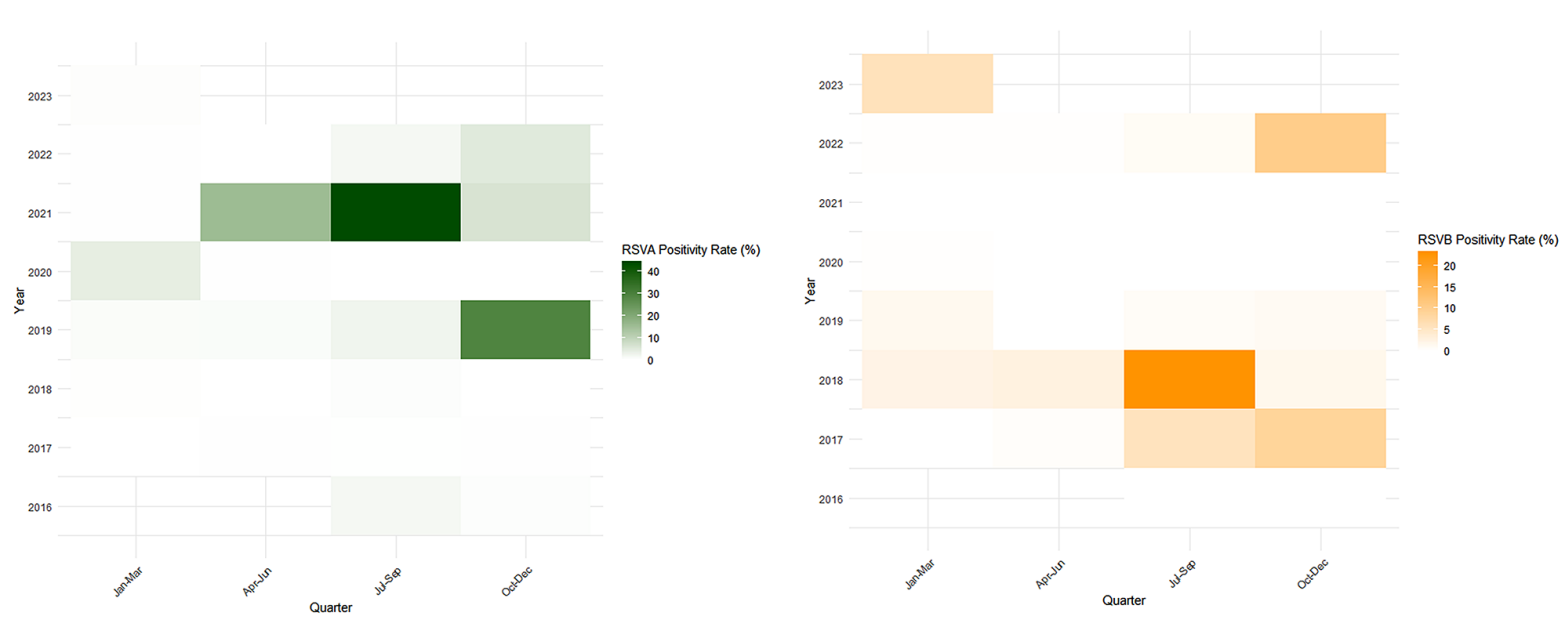
Heat-map showing quarter-wise activity of RSV-A and RSV-B from 2016–2023

Figure 2. In the pre-pandemic period, the RSVA virus maintained a relatively low level of prevalence, with a significant spike in the fourth quarter of 2019, marking a positivity rate of 28.9%. This peak notably coincided with the colder months and higher precipitation, suggesting a potential link between the virus’s activity and the weather conditions. Upon the onset of the pandemic, the virus activity varied dramatically. The latter half of 2020 recorded a 0% positivity rate, while a surge in the third quarter of 2021 saw the rate reaching an unprecedented high of 44.1% See fig. 3.

Similarly, the positivity rate for the RSVB virus varied throughout different quarters across both the periods. Remarkably, the virus presents no cases in several quarters as suggested by the zero-positivity rate. However, in specific quarters like July to September in 2018 and October to December in 2022, we notice a significant surge in the positivity rate, escalating to as high as 23.4% and 10.4% respectively. It is noteworthy that the higher activity of the RSV A and RSVB virus appears to align with quarters known for higher precipitation and cooler months. Table 2 During the pre-pandemic years, the most substantial co-infection rates with RSV were seen with Rhinovirus (3.8%), Influenza A and H1N1pdm09 (both at 2.8%), and Adenovirus (2.0%). Contrastingly, the pandemic years presented a general decline in co-infection rates. In 2020, no co-infections of RSV with any of the listed viruses were reported. The year 2021 showed the most prominent co-infection rates with Influenza B and H3N2, standing at 2.6% and 2.1%, respectively. It’s also notable that 2021 marked the debut of a 0.5% co-infection rate of RSV with SARS CoV-2. In 2022, the highest co-infection rate with RSV was observed with Rhinovirus at 2.4%, followed by Adenovirus at 1.9%. For the most recent data available from 2023, Influenza A, H3N2, and Adenovirus all exhibit the highest co-infection rates with RSV at 3.5%.

**Table 2:** Comparison of RSV Co-infection

Co-infection with RSV	Pre-pandemic (2016-Jan2020)	Pandemic 2020	Pandemic 2021	Pandemic 2022	Pandemic 2023
	RSV Positive	RSV Positive	RSV Positive	RSV Positive	RSV Positive
	*N* = 398	*N* = 16	*N* = 341	*N* = 212	*N* = 85
Influenza A	11 (2.8%)	0 (0%)	8 (2.3%)	3 (1.4%)	3 (3.5%)
influenza B	3 (0.8%)	0 (0%)	9 (2.6%)	0 (0%)	1 (1.2%)
SARS CoV-2	0 (0%)	0 (0%)	0 (0%)	1 (0.5%)	0 (0%)
Inf A(H1N1) pdm09	11 (2.8%)	0 (0%)	1 (0.3%)	2 (0.9%)	0 (0%)
Inf A (H3N2)	0 (0%)	0 (0%)	7 (2.1%)	1 (0.5%)	3 (3.5%)
HMPV	3 (0.8%)	0 (0%)	1 (0.3%)	2 (0.9%)	0 (0%)
PIV1	0 (0%)	0 (0%)	0 (0%)	0 (0%)	0 (0%)
PIV2	1 (0.3%)	0 (0%)	0 (0%)	0 (0%)	0 (0%)
PIV3	5 (1.3%)	2 (12%)	2 (0.6%)	1 (0.5%)	1 (1.2%)
PIV4	1 (0.3%)	0 (0%)	0 (0%)	0 (0%)	0 (0%)
Adenovirus	8 (2.0%)	0 (0%)	4 (1.2%)	4 (1.9%)	3 (3.5%)
Rhinovirus	15 (3.8%)	0 (0%)	1 (0.3%)	5 (2.4%)	1 (1.2%)

## Discussion

The COVID-19 pandemic introduced unique dynamics to the spread and prevalence of other infectious diseases, including Respiratory Syncytial Virus (RSV). Comparing RSV data between pre-pandemic years and pandemic years, several noteworthy patterns emerge. One of the most striking findings from our study is the dramatic decrease in RSV positivity rate in 2020, from an average 7.2% in pre-pandemic years to 1.5%. This drastic drop is likely attributable to widespread implementation of mitigation measures such as social distancing, mask-wearing, and enhanced sanitation to combat the COVID-19 pandemic, which simultaneously impeded the transmission of other respiratory viruses like RSV. This supports findings by Kuitunen et al. (2021), which indicated a decline in RSV infections during the COVID-19 lockdowns [14]. However, an interesting rebound was observed in 2021, where RSV positivity rate surged to 32.0%. This unexpected surge may be due to resumption of unrestricted social interaction and schooling in 2021 created opportunities for RSV to spread in communities that had built up little immunity over the previous year, as also reported by Foley et al. (2021) in their Australian study [4]. Another key observation pertains to the age distribution of RSV cases. Both during the pre-pandemic and pandemic periods, the highest number of RSV cases was in the 0–4 years age group, reflecting the known vulnerability of this age group to RSV as per Hall et al. (2009) [15]. And among them less than two years of age made the majority of the cases, a separate manuscript exploring the RSV in pediatric cases will be discussed elsewhere. RSV is notorious for causing severe respiratory illness in young children, particularly those under two years of age [3, 16]. The surge in RSV hospitalizations in 2021 points to the need for continued protective measures to prevent overwhelming pediatric healthcare systems during peak RSV activity periods. Additionally, it underscores the role of routine RSV surveillance in enabling quick responses to changes in trend, aligning with WHO recommendations [17]. The observed data show considerable variation in the detection rates of Respiratory Syncytial Virus A (RSVA) and B (RSVB) during both pre-pandemic and pandemic years. These patterns may be influenced by several factors, including the natural cyclical nature of respiratory syncytial viruses, the impact of public health measures to limit the spread of COVID-19, and variations in testing. In the pre-pandemic period, both RSVA and RSVB exhibited fluctuating annual detection rates. RSVA accounted for a modest proportion of the total cases each year, ranging from 0.3% in 2016 to a peak of 23% in 2019. The higher prevalence of RSVB in comparison to RSVA, except in 2019, corresponds with the observations from several previous studies that suggest alternate circulation of the two subtypes in many regions, possibly due to immune selective pressure or other ecological factors (Peret et al., 1998; Tang et al., 2017) [18, 19]. The sudden drop in the prevalence of both RSVA and RSVB in 2020 aligns with the global implementation of public health measures to limit the spread of COVID-19. (Baker et al., 2020) [1]. The significant surge in RSVA cases in 2021, without the detection of RSVB, is noteworthy the absence of RSVB during this period could be due to the alternate circulation of the subtypes or could be impacted by other unknown factors. This needs to be further studied and understood (Shi et al., 2020) [3]. The year 2022 witnessed a reduction in RSVA cases and a resurgence of RSVB, possibly reflecting the natural cyclical pattern of these two subtypes. This phenomenon has been previously reported where circulation of one subtype is followed by the dominance of the other subtype in subsequent years (Anderson et al., 2013) [20]. The lack of significant gender disparity in RSV cases in both periods is consistent with a large body of literature, which suggests that susceptibility to RSV is not gender-dependent [21]. The pandemic also impacted the clinical management of RSV. Notably, there was a drastic reduction in the use of antivirals and ventilator support during the pandemic years compared to the pre-pandemic period. This could be attributed to global efforts to preserve resources for COVID-19 patients, coupled with lower RSV disease severity due to social distancing and mask-wearing measures. Lastly, the co-infection rates of RSV with other viruses notably declined during the pandemic, which could be related to reduce circulation of other respiratory viruses due to COVID-19 mitigation measures. Another aspect to consider is the ‘viral interference’ phenomenon, where the presence of one virus can inhibit the replication or affected the spread of another [22]. During the pandemic, the dominance of COVID-19 could have played a role in suppressing the circulation of other respiratory viruses, including those that typically co-infect with RSV. The major limitation of our study is that it was confined to a single healthcare network and a limited geographic region. Larger, multi-center studies across diverse populations would strengthen the evidence base for the resurgence of RSV following relaxation of COVID-19 containment measures. Further research is also needed to determine optimal strategies for controlling the spread of RSV as societies open up, including the continued targeted use of masks, social distancing and hand hygiene [3, 23]. In our study we used different swab collection method as per the patient age, iIn younger children, the nasopharyngeal area is a common site for viral colonization, making nasal swabs a practical choice for viral detection. Respiratory viruses, including respiratory syncytial virus (RSV), influenza, and the common cold viruses, can be effectively detected using this method. For individuals older than 5 years, including both throat and nasal swabs can enhance the sensitivity of viral detection. Some viruses may preferentially infect the throat or can be present in higher concentrations in the throat than in the nasal passages. Collecting both nasal and throat swabs in older patients can increase the likelihood of detecting viral pathogens, as it covers more potential sites of infection. However still, the quality and quantity of specimens collected can vary significantly between nasal and throat swabs, and among different age groups, due to differences in technique.

However, study provided insight into as to how, a resurgence of RSV cases was observed in multiple regions when the pandemic situation improved and restrictions were eased. This rebound in RSV infections can be attributed to several factors. First, the relaxation of public health measures may have led to increased interpersonal contact and, consequently, a greater likelihood of RSV transmission [24]. Second, the decreased exposure to RSV during the pandemic might have resulted in a susceptible population, particularly among young children who have not yet developed immunity against RSV [25]. Third, the timing of RSV resurgence appears to coincide with the reopening of schools and childcare facilities, which are known to be hotspots for RSV transmission [23]. This resurgence of RSV has several implications for public health and healthcare systems. The increased burden of RSV cases may strain healthcare resources and lead to a rise in hospitalizations and severe cases, particularly among vulnerable populations such as infants, young children, and the elderly [26]. Additionally, the resurgence of RSV may complicate the diagnosis and management of respiratory illnesses, as symptoms of RSV infection can overlap with those of COVID-19 and other respiratory viruses [12]. This necessitates the need for comprehensive and accurate diagnostic testing to differentiate between various respiratory pathogens and inform appropriate clinical management [27].

In summary, this comparison reveals the significant and multifaceted impact of the COVID-19 pandemic on RSV epidemiology, clinical manifestations, and management. Further research is warranted to better understand these changes and inform public health strategies for managing RSV in the evolving context of COVID-19 and beyond.

## Data Availability

The datasets generated during and analyzed during the current study are not publicly available but are available from the corresponding author on reasonable request.

## References

[1] Baker RE, Park SW, Yang W, Vecchi GA, Metcalf CJE, Grenfell BT (2020). The impact of COVID-19 nonpharmaceutical interventions on the future dynamics of endemic infections. Proc Natl Acad Sci.

[2] Sawicki GS, Sawicki DL (2021). Respiratory syncytial virus in the era of COVID-19. J Clin Virol.

[3] Shi T, McAllister DA, O’Brien KL, Simoes EAF, Madhi SA, Gessner BD, Campbell H (2017). Global, regional, and national disease burden estimates of acute lower respiratory infections due to respiratory syncytial virus in young children in 2015: a systematic review and modelling study. Lancet.

[4] Foley DA, Yeoh DK, Minney-Smith CA, Martin AC, Mace AO, Sikazwe CT, Le H, Levy A, Blyth CC, Moore HC (2021). The interseasonal resurgence of respiratory syncytial virus in Australian children following the reduction of coronavirus disease 2019-related public health measures. Clin Infect Dis.

[5] Britton PN, Hu N, Saravanos G, Shrapnel J, Davis J, Snelling T, Dalby-Payne J, Kesson A, Wood N, Macartney K (2021). COVID-19 public health measures and the indirect impact on the incidence of childhood respiratory tract infections, sepsis and antibiotic use in Australia: an interrupted time series analysis. Lancet Child Adolesc Health.

[6] Glezen WP (2021). The changing epidemiology of respiratory syncytial virus disease: implications for the use of palivizumab. Pediatr Infect Disease J.

[7] Kim JH, Park JS, Lee JH (2021). Respiratory syncytial virus in the era of coronavirus disease 2019 (COVID-19). Infect Chemother.

[8] Blanken MO, Rovers MM, Molenaar JM, Winkler-Seinstra PL, Meijer A, Kimpen JL, Bont L (2021). Respiratory syncytial virus and SARS-CoV-2 co-infection in hospitalized children. J Infect.

[9] Rha B, Curns AT, Lively JY, Campbell AP, Englund JA, Boom JA, Weinberg GA (2021). Respiratory syncytial virus-associated hospitalizations—United States, July 2020–March 2021. Morb Mortal Wkly Rep.

[10] Chotpitayasunondh T, Fischer TK, Poovorawan Y (2021). Respiratory Syncytial Virus infections in Southeast Asia. Pediatr Infect Disease J.

[11] Thongpan I, Vichaiwattana P, Klinfueng S, Wongsrisang P, Theamboonlers A, Poovorawan Y. (2021). Prevalence of human respiratory syncytial virus and human metapneumovirus during the COVID-19 pandemic in Thailand. PLoS ONE, 16(11), e0259790.

[12] Naing ZW, Chidlow GR, Smith DW (2022). Increased detection of respiratory syncytial virus and other respiratory viruses in Western Australia after the relaxation of COVID-19 control measures. Pathology.

[13] Chadha M, Prabhakaran AO, Choudhary ML, Biswas D, Koul P (2022). Multisite surveillance for influenza and other respiratory viruses in India: 2016–2018. PLOS Global Public Health.

[14] Kuitunen I, Artama M, Mäkelä L, Backman K, Heiskanen-Kosma T, Renko M (2020). Effect of social distancing due to the COVID-19 pandemic on the incidence of viral respiratory tract infections in children in Finland during early 2020. Pediatr Infect Dis J.

[15] Hall CB, Weinberg GA, Iwane MK, Blumkin AK, Edwards KM, Staat MA, Auinger P, Griffin MR, Poehling KA, Erdman D, Grijalva CG, Zhu Y, Szilagyi P (2009). The burden of respiratory syncytial virus infection in young children. N Engl J Med.

[16] Hall CB, Weinberg GA, Iwane MK (2013). Respiratory syncytial virus-associated hospitalizations among children less than 24 months of age. Pediatrics.

[17] World Health Organization. Immunization, Vaccines and Biologicals: Respiratory Syncytial Virus. https://www.who.int/immunization/diseases/RSV/en/.

[18] Peret TCT (1998). Circulation patterns of genetically distinct group A and B strains of human respiratory syncytial virus in a community. J Gen Virol.

[19] Tang JW (2017). Global epidemiology of non-influenza RNA respiratory viruses: data gaps and a growing need for surveillance. Lancet Infect Dis.

[20] Anderson LJ, Garg S, Whitaker M, O’Halloran A et al. Hospitalization Rates and Characteristics of Patients Hospitalized with Laboratory-Confirmed Coronavirus Disease 2019 — COVID-NET, 14 States, March 1–30, 2020. MMWR Morb Mortal Wkly Rep 2020;69:458–464. 10.15585/mmwr.mm6915e3. 10.15585/mmwr.mm6915e3 PMC7755063 32298251

[21] Grijalva CG, Rolfes MA, Zhu Y (2020). Transmission of SARS-COV-2 infections in households — Tennessee and Wisconsin, April–September 2020. MMWR Morb Mortal Wkly Rep.

[22] Dianzani F. Viral interference and interferon. Ric Clin Lab. 1975 Jul-Sep;5(3):196–213. 10.1007/BF02908284. PMID: 778995. 10.1007/BF02908284 778995

[23] Shi T, McAllister DA, O’Brien KL (2017). Global, regional, and national disease burden estimates of acute lower respiratory infections due to respiratory syncytial virus in young children in 2015: a systematic review and modelling study. Lancet.

[24] Kim L, Rha B, Abramson JS (2022). Resurgence of respiratory Syncytial Virus infections during COVID-19 pandemic, South Korea. Emerg Infect Dis.

[25] Friedrich F, Ongaratto R, Scotta MC (2021). Early Impact of Social Distancing in response to COVID-19 on hospitalizations for Acute Bronchiolitis in infants in Brazil. Clin Infect Dis.

[26] Poole S, Brendish NJ, Tanner AR, Clark TW (2020). Physical distancing in schools for SARS-CoV-2 and the resurgence of rhinovirus. Lancet Respir Med.

[27] Charlton CL, Babady E, Ginocchio CC (2019). Practical Guidance for Clinical Microbiology Laboratories: viruses causing Acute respiratory tract infections. Clin Microbiol Rev.

